# *Bacillus thuringiensis* CbpA is a collagen binding cell surface protein under c-di-GMP control

**DOI:** 10.1016/j.tcsw.2019.100032

**Published:** 2019-08-23

**Authors:** Sarah Finke, Annette Fagerlund, Veronika Smith, Veronica Krogstad, Mimmi Jingxi Zhang, Athanasios Saragliadis, Dirk Linke, Christina Nielsen-LeRoux, Ole Andreas Økstad

**Affiliations:** a Centre for Integrative Microbial Evolution and Section for Pharmaceutical Biosciences, Department of Pharmacy, University of Oslo, Norway; b Department of Biosciences, University of Oslo, Norway; c INRA UMR1319 Micalis AgroParisTech, Paris Saclay University, Jouy-en-Josas, France

**Keywords:** *Bacillus cereus* group, Cyclic-di-GMP, Adhesion, Collagen binding

## Abstract

Cyclic diguanylate (c-di-GMP) signalling affects several cellular processes in *Bacillus cereus* group bacteria including biofilm formation and motility, and CdgF was previously identified as a diguanylate cyclase promoting biofilm formation in *B. thuringiensis.* C-di-GMP can exert its function as a second messenger via riboswitch binding, and a functional c-di-GMP-responsive riboswitch has been found upstream of *cbpA* in various *B. cereus* group strains. Protein signature recognition predicted CbpA to be a cell wall-anchored surface protein with a fibrinogen or collagen binding domain. The aim of this study was to identify the binding ligand of CbpA and the function of CbpA in cellular processes that are part of the *B. cereus* group c-di-GMP regulatory network. By global gene expression profiling *cbpA* was found to be down-regulated in a *cdgF* deletion mutant, and *cbpA* exhibited maximum expression in early exponential growth. Contrary to the wild type, a *ΔcbpA* deletion mutant showed no binding to collagen in a cell adhesion assay, while a CbpA overexpression strain exhibited slightly increased collagen binding compared to the control. For both fibrinogen and fibronectin there was however no change in binding activity compared to controls, and CbpA did not appear to contribute to binding to abiotic surfaces (polystyrene, glass, steel). Also, the CbpA overexpression strain appeared to be less motile and showed a decrease in biofilm formation compared to the control. This study provides the first experimental proof that the binding ligand of the c-di-GMP regulated adhesin CbpA is collagen.

## 1 Introduction

The *Bacillus cereus* group consists of at least seven different species of bacteria, and an additional fourteen species have recently been proposed, on the basis of a wide set of different criteria (Peak et al., 2007, Jung et al., 2010, Jung et al., 2011, Jimenez et al., 2013, Miller et al., 2016, Liu et al., 2017). The group encompasses *Bacillus anthracis*, an obligate pathogen of herbivores which also causes life-threatening disease in humans, most often through contact with infected animals or animal products - and also includes *Bacillus cereus*, an opportunistic human pathogen responsible for emetic or diarrhoeal syndromes (strain dependent), but also a range of other diseases in immunocompromised individuals (reviewed in Drobniewski, 1993, Bottone, 2010). *Bacillus thuringiensis* is phylogenetically intermixed with *B. cereus*, and is classified through its entomopathogenicity, more specifically its ability to kill insect larvae, through the synthesis of insecticidal protein toxins which are often encoded on extrachromosomal elements (Nielsen-LeRoux et al., 2012). *B. thuringiensis*, although primarily having been regarded a pathogen of insects, carries the same set of chromosomal virulence factors as *B. cereus*, several of which are known to be linked to virulence also during human infection (Ngamwongsatit et al., 2008)*. B. cereus* and *B. thuringiensis* are ubiquitously present in the environment, in soil, air and water, and, analogous to *B. anthracis* which has a life cycle involving fulminant infection of herbivores and the soil/vegetation niche, have a complex ecological cycle which may involve cycling between the gut of insect larvae and the soil/plant environment (Jensen et al., 2003, Saile and Koehler, 2006, Ceuppens et al., 2013, Vidal-Quist et al., 2013).

The complex life styles of these bacteria require an adaptive mode of global gene regulation, enabling the transition between different hosts and enduring highly diverse environmental niches to which the bacteria may be exposed when being shed from a host. *B. thuringiensis* harbours key transcriptional regulators involved in governing the infectious cycle in the insect – PlcR constituting a key activator of virulence genes promoting spread of the bacterium within the insect during active infection (Gohar et al., 2008) and NprR governing necrotrophism, the survival of the bacterium following the death of the insect host (Dubois et al., 2012).

*B. cereus* group bacteria, similar to *Bacillus subtilis,* have the capability of forming biofilms – a process regulated by the transcriptional regulator SinR, a repressor of biofilm matrix genes (Pflughoeft et al., 2011, Fagerlund et al., 2014). Biofilm formation is also controlled by the second messenger cyclic di-GMP (c-di-GMP), which has been shown in a range of bacterial species to be involved in regulating the switch between a motile and sessile lifestyle (e.g. reviewed in Jenal et al., 2017). Most *B. cereus* group strains carry a complement of diguanylate cyclases and phosphodiesterases, which are enzymes involved in c-di-GMP synthesis and breakdown, respectively (Fagerlund et al., 2016). One of the highly conserved c-di-GMP metabolism genes is *cdgF*, which encodes a dual function protein capable of acting as a diguanylate cyclase (GGDEF domain) or a phosphodiesterase (EAL domain), depending on its red-ox status (Fagerlund et al., 2016).

Adhesion, whether to biotic or abiotic surfaces, constitutes the initial step of biofilm formation, and is also a key step during the infectious process for many human and animal pathogens, enabling colonisation of the host. Collagen and fibrinogen may constitute ligands for bacterial surface adhesins governing these initial steps of the infectious process. Here we identify, by global transcriptional profiling of a *cdgF* deletion mutant (Fagerlund et al., 2016), a putative cell surface adhesion protein in *B. thuringiensis* 407 (*cry-*) which is under c-di-GMP transcriptional control and linked to CdgF function (Lee et al., 2010, Tang et al., 2016). We perform a thorough characterisation of the protein and establish the first binding ligand identification for a c-di-GMP effector protein in the *B. cereus* group, constituting the first step towards unravelling a c-di-GMP responsive network for a *B. cereus* group bacterium.

## 2 Methods

### 2.1 Bioinformatics analysis

At the time of analysis 144 fully sequenced and closed genomes of members of the *B. cereus* group were available from the NCBI (https://www.ncbi.nlm.nih.gov/). Their genomes and proteomes were searched with the *cbpA*/CbpA sequence from *B. thuringiensis* 407 using BlastN and BlastP. InterProScan (Jones et al., 2014) was utilized for prediction of protein domains and Illustrator for Biological Sequences (IBS) was used to illustrate protein domain structure (Liu et al., 2015). The web-application Riboswitch Scanner (Singh et al., 2009, Mukherjee and Sengupta, 2016) (http://service.iiserkol.ac.in/~riboscan/application.html) was used to explore DNA sequences for class I c-di-GMP riboswitches.

### 2.2 Phylogenetic tree construction

Altogether 113 strains were used to build a phylogenetic tree representative of the *B. cereus* group. The tree was constructed as in Fagerlund et al. (2016) by employing the distance based Neighbour-Joining method BioNJ (Gascuel, 1997) on MLST data obtained using the Tourasse-Helgason MLST scheme (Helgason et al., 2004) (http://mlstoslo.uio.no). Tree construction and visualization was executed using Seaview 4 (Gouy et al., 2010) and TreeGraph 2 (Stover and Muller, 2010).

### 2.3 Bacterial strains and culture conditions

The model strain used in this study is the acrystalliferous *B. thuringiensis* strain 407 Cry^-^ (Lereclus et al., 1989, Sheppard et al., 2013). Cultures were routinely inoculated to an optical density at 600 nm (OD_600_) of 0.05 from an overnight culture and grown at 30 ͦC and 220 rpm in Luria Bertani (LB) broth or bactopeptone medium (1% w/v bactopeptone, 0.5% w/v yeast extract, 1% w/v NaCl). For cloning and expression in *Escherichia coli*, ampicillin was used at 50 or 100 µg ml^−1^, kanamycin at 50 µg ml^−1^, erythromycin at 200 µg ml^−1^ (pMAD), and tetracycline at 12 µg ml^−1^. Erythromycin was used at 10 µg ml^−1^ to maintain the pHT304-Pxyl plasmid constructs in *B. thuringiensis*. Unless otherwise indicated, induction of gene expression from the *xylA* promoter in pHT304-Pxyl was performed by addition of 1 mM xylose to the growth medium.

### 2.4 Construction of deletion mutants and overexpression strains

For overexpression and complementation, the low-copy number *E. coli*/*Bacillus* shuttle vector pHT304-Pxyl was used, in which *xylR* and the *xylA* promoter from *B. subtilis* was inserted into the pHT304 cloning site (Arantes and Lereclus, 1991), allowing xylose-inducible expression of downstream cloned genes. The *cbpA* gene was PCR amplified from *B. thuringiensis* 407 using primers listed in Supplementary Table S1, and inserted into the plasmid vector using primer-incorporated restriction sites.

The Δ*cbpA* mutant was generated via homologous recombination using a markerless gene replacement method (Janes and Stibitz, 2006). To enable facilitated detection of transformants using blue-white screening on X-gal (5-bromo-4-chloro-3-indolyl-β-D-galactopyranoside) plates, the pMAD-I-*Sce*I shuttle vector (Arnaud et al., 2004, Fagerlund et al., 2016) carrying a constitutively expressed β-galactosidase gene and an I-*Sce*I restriction site, was used as the integrative plasmid.

The mutant allele was designed to contain the start and stop codons of the deleted *cbpA* gene, separated by the CAATTG recognition sequence for restriction enzyme *Mfe*I, thus creating an in-frame deletion not expected to exert polar effects on surrounding genes. Approximately 700 bp of DNA sequence homologous to the upstream and downstream region of the gene was PCR amplified using primers listed in Supplementary Table S1. The up- and downstream fragments were fused using the primer-incorporated restriction sites, cloned into pCRII-TOPO vector (Invitrogen) and then transferred to pMAD-I-*Sce*I. The constructs were introduced into *B. thuringiensis* 407 by electroporation (Masson et al., 1989) and allelic exchange was performed essentially as described (Janes and Stibitz, 2006). All mutant alleles were verified by sequencing of PCR products generated with primers designed to anneal outside of the sequences used for homologous recombination (Supplementary Table S1). Plasmid and strains used in this study are listed in Table 1.

**Table 1:** Strains and plasmids used in this study. ^a^Ap^r^, Ery^r^, Tet^r^: resistance to ampicillin, erythromycin, tetracycline, respectively.

Strain or plasmid	Relevant characteristics/Description^a^	Reference or source
**Plasmids**		
pCRII–TOPO	TOPO vector for cloning of *cbpA*	Invitrogen
pMAD-I-SceI	Plasmid used for making deletion mutants, containing I-SceI restriction site, Ap^r^, Ery^r^	(Arnaud et al., 2004, Fagerlund et al., 2016)
pMAD-*cbpA*	Plasmid pMAD-I-SceI, containing elements flanking *cbpA*, used for creating markerless *cbpA* deletion mutant, Ap^r^, Ery^r^	This study
pBKJ223	Plasmid encoded homing restriction enzyme I-*Sce*I, promoting the second homologous recombination event in procedure for making markerless deletion mutant, Tet^r^	(Janes and Stibitz 2006)
pHT304-Pxyl	Low copy number *E. coli*/*Bacillus* shuttle and expression vector; *xylA*+; *xylA* promoter; Ap^r^, Ery^r^	(Arantes and Lereclus 1991)
pHT304-Pxyl-*cbpA*	Plasmid used for xylose-inducible expression of *cpbA*; Ap^r^, Ery^r^	This study

**Strains**		
***Escherichia coli***		
XL1-Blue MRF’	Cloning strain, Tet^r^	Agilent
TOP10	Cloning strain	Invitrogen

***B. cereus* group**		
Bt407	*B. thuringiensis* 407 *cry*^-^	(Lereclus et al., 1989, Sheppard et al., 2013)
Bt407 pHT304-Pxyl	Bt407 containing pHT304-Pxyl empty vector; Ery^r^, vector control strain	(Fagerlund et al., 2016)
Bt407 pHT304-Pxyl-*cbpA*	Bt407 overexpressing CbpA; Ery^r^	This study
Bt407Δ*bspA*	Bt407 with *in frame* markerless deletion of *cbpA*	This study
Bt407Δ*bspA* pHT304-Pxyl-*cbpA*	Bt407Δ*cbpA* expressing CbpA from the pHT304-Pxyl plasmid (complemented mutant); Ery^r^	This study
Bt407Δ*cdgF*	Bt407 with *in frame* markerless deletion of *cdgF*	(Fagerlund et al., 2016)

### 2.5 Isolation of RNA and reverse transcription quantitative PCR

For the isolation of RNA from biofilm and planktonic cells for subsequent RT-qPCR, methods described in Fagerlund et al. (2016) were followed. Briefly, cells were allowed to form biofilm on glass wool and samples were harvested after 24 h incubation by transfer of biofilm cells to ice-cold 60% methanol. Planktonic cells were incubated with an equal volume of ice-cold methanol and all samples were centrifuged for collection of cells. Cell lysis was done using a Precellys 24 Tissue Homogenizer (Bertin) and RNeasy Mini or Midi Kits (Qiagen) were used for RNA isolation. After treatment with DNase and further purification, cDNA synthesis was performed with SuperScript III Reverse Transcriptase (Invitrogen), performed in duplicate for each sample (two technical replicates). For all samples a negative control reaction without reverse transcriptase was included. RT-qPCR was carried out in a LightCycler 480 Real-Time PCR System (Roche) using primers shown in Supplementary Table S1. The second derivative maximum method in the LightCycler 480 software (Roche) was utilized to obtain quantification cycle (C_q_) values. The expression of the target gene in each biological replicate was converted into E^Cq^ values (Pfaffl, 2001) and then normalized to the geometric mean of the E^Cq^ values determined for the three reference genes *gatB/yqeY*, *rpsU*, and *udp* (Reiter et al., 2011).

### 2.6 Global transcriptional profiling using 70-mer oligonucleotide microarrays

RNA isolation, cDNA synthesis, labeling and purification was performed as previously described (Gohar et al., 2008). RNA was isolated from *B. thuringiensis* 407 cells in exponential growth (1.5 h after inoculation) and was precipitated (20 μg) in 0.3 M NaAc (pH 5.5) and 70% ethanol overnight at −80 °C ahead of cDNA preparation. Microarray slides were printed at The microarray core facility of the Norwegian University of Science and Technology (NTNU). Design, printing, prehybridization, hybridization and scanning of the slides, and analysis of the data, was performed as described in Gohar et al. (2008). The microarray experiment was based on four slides, all biological replicates. P-values were computed using a false discovery rate (FDR) of 0.05.

The microarray slides contain 70-mer oligonucleotide probes designed to detect open reading frames (ORFs) in *B. anthracis* Ames, *B. anthracis* A2012, and *B. cereus* ATCC 14579, in addition to selected genes from *B. cereus* ATCC 10987 (Kristoffersen et al., 2007). To facilitate analyses in *B. thuringiensis* 407, all probe sequences on the microarray had been analyzed by BLAST for hits to annotated genes in the *B. thuringiensis* 407 draft genome sequence (GenBank accession ACMZ00000000.1, as of 30.04.2009), and the gene lists used for microarray analysis are based on the annotations from this GenBank entry. Only probes with 93% identity or greater to a transcript/feature sequence of *B. thuringiensis* 407 were included in the analysis. COG categories were obtained for the analyzed genes as reported in the IMG database (http://img.jgi.doe.gov). The data obtained from this analysis was submitted to the ArrayExpress archive (https://www.ebi.ac.uk/arrayexpress/) under the accession number E-MTAB-8092.

### 2.7 Biofilm assay

Biofilm forming capability was investigated in a multi-well plate screening assay, modified from a previously described method (Auger et al., 2006). Cultures grown to early exponential phase in bactopeptone medium at 30 °C (OD_600_ ≈ 0.3) were used to inoculate fresh bactopeptone medium to an OD_600_ of 0.01. For each strain, four wells of a 24-well polystyrene plate (Falcon 353047) were filled with 500 µl of the bacterial culture suspension. Plates were produced in duplicate, and each plate contained four wells of bactopeptone medium as control. Following incubation at 30 ͦC for 24 h, 48 h and 72 h, the wells of one of the microplates were washed once with phosphate-buffered saline (PBS) and stained with an 0.1% (w/v) aqueous solution of methyl violet 6B for 30 min at room temperature. The wells were then washed three times with PBS and dried upside down over night. For quantification of biofilm formation, the dye was solubilized by adding 500 µl of a 1:4 acetone/ethanol mixture to each well and incubated for 10 min at room temperature, followed by measuring the absorbance at 575 nm in a plate reader (BMG Labtech ClarioStar).

### 2.8 Motility assay

Motility was assessed essentially as described in Fagerlund et al. (2016). Bacteria were grown in LB medium to OD_600_ ≈ 1.0, and 5 µl of culture was spotted on LB agar plates containing 0.3% agar, with 1 mM xylose and/or 10 µg ml^−1^ erythromycin added if appropriate. Plates were incubated for 7 h at 30 °C and motility was measured as the distance between the colony edge and the outer line of the swimming zone.

### 2.9 Whole cell adhesion assay

Binding to biotic surfaces was investigated with a whole cell adhesion assay adapted from the method described in Saragliadis and Linke (2019). The following substances (purchased from Sigma-Aldrich and Merck) were used in the assay: Collagen type I (calf skin, C9791), collagen type II (bovine cartilage, C1188), collagen type III (human placenta C4407), collagen type IV (human placenta, C7521), collagen type V (human plasma, C3657), fibrinogen (bovine, 341573), fibronectin (bovine plasma, F4759). All collagens were diluted to 20 µg ml^−1^ in 0.01 M acetic acid, while fibrinogen and fibronectin were diluted in PBS. 96-well polystyrene microplates (Corning 3370) were coated with 125 µl per well of each substance for a duration of 1 h at room temperature. After blocking with 50 mg ml^−1^ bovine serum albumin (BSA, Sigma-Aldrich) in PBS for 30 min at room temperature, the wells were washed three times with 0.1 mg ml^−1^ BSA in PBS. Ahead of the assay, bacteria were grown to early exponential phase at 30 ͦC (OD_600_ ≈ 0.2–0.3) in LB medium, supplemented with 10 µg ml^−1^ erythromycin and 1 mM xylose for strains containing the pHT304-Pxyl plasmid. Bacterial culture suspensions were centrifuged for 15 min at 1500×*g*, resuspended in PBS and adjusted to an OD_600_ of 0.9–1.0. For each strain in each independent experiment, eight collagen-coated wells and eight empty wells, representing technical replicates, were filled with 125 µl of the bacterial suspension and incubated for 1 h at room temperature. Additionally, eight collagen-coated and eight empty wells (technical replicates) were incubated with PBS as negative controls. After washing three times with PBS, wells were stained with 125 µl of an 0.1% (w/v) aqueous solution of methyl violet 6B for 30 min at room temperature. Solubilization of the dye was performed as described for the biofilm assay, but with 125 µl 1:4 acetone/ethanol mixture. Absorbance at 575 nm was measured in a plate reader (BMG Labtech ClarioStar).

### 2.10 Adhesion to abiotic surfaces

For examination of binding to abiotic surfaces, bacteria were grown and prepared as described above (2.9). Steel coupons were prepared as described earlier (Castelijn et al., 2013) by treatment with 1 M NaOH at 50 °C for 30 min, followed by washing with dH_2_O and incubation in acetone for 15 min at room temperature. After four subsequent washes in dH_2_O the steel coupons were autoclaved. Glass cover slips were autoclaved and all plates were dried overnight. The adhesion assay is based on a method described by Hayrapetyan and co-workers (Hayrapetyan et al., 2015). Coupons and plates were placed vertically into the wells of 12-well plates (Corning 3737) containing 2 ml bacterial suspension per well. After 60 min of incubation at room temperature, coupons and plates were washed by gently dipping them into sterile PBS. Each coupon was then transferred to a 50 ml tube containing 3 ml sterile PBS and 0.5 g autoclaved glass beads (Sigma-Aldrich G4649 (<106 µm)). To detach cells from the coupons, tubes were vortexed at full speed for 1 min. The number of colony forming units (CFUs) for each coupon was determined from this bacterial suspension.

### 2.11 *Galleria mellonella in vivo* infection assay

The virulence-related properties of CbpA were assessed by comparing the killing effect of the *B. thuringiensis* 407 wild type (Bt407), the *cbpA* deletion strain (Bt407Δ*cbpA*), and the complementation strain (Bt407Δ*cbpA* pHT304-Pxyl-*cbpA*), by infection (force feeding) in 5th instar *Galleria mellonella* larvae. *G. mellonella* eggs were hatched at 28 °C and the larvae reared on beeswax and pollen. For infection experiments, groups of 20 to 25 *G. mellonella* larvae, weighing about 200 mg were used. Xylose (20 mM) was added to the LB growth medium of all strains, as well as to the bacterial inoculums and the toxin alone control (Cry1C) at time zero (time point of force feeding). Larvae were force fed a second time with 10 µl 20 mM xylose 5 h later (in order to again activate CbpA expression from the pHT304-Pxyl plasmid in the complementation strain). Infections were otherwise performed as previously described (Fedhila et al., 2006) by force feeding larvae with 10 µl of a mixture containing 4–5 × 10^6^ of vegetative bacteria (exponential growth OD_600_ ≈ 1 in LB with 20 mM xylose) and 3 µg of activated Cry1C toxin. The larvae in the control group were fed either 10 μl PBS buffer or 10 μl Cry1C toxin + xylose. Mortality of the infected larvae was observed after 4 h, 24 h and 48 h. The chosen dose was expected to result in about 70 (±5) % larvae mortality for the infection with the wild type *B. thuringiensis* 407 at 37 °C after 48 h (Ramarao et al., 2012).

## 3 Results

### 3.1 Transcriptomic analysis reveals that *cbpA* expression is influenced by cellular c-di-GMP levels

Species in the *B. cereus* group have several genes encoding proteins with GGDEF and/or EAL domains (Fagerlund et al., 2016) predicted to be involved in metabolism of c-di-GMP. In the biofilm model strain *B. thuringiensis* 407, belonging to phylogenetic group IV within the *B. cereus* group (Guinebretiere et al., 2008), eleven genes encoding GGDEF and/or EAL domains have been identified, of which eight were predicted or shown to have c-di-GMP metabolizing enzymatic activity. Among these, the tandem GGDEF-EAL protein CdgF was shown to be the main c-di-GMP metabolizing enzyme controlling biofilm formation in *B. thuringiensis* 407 under oxygenated conditions, and biofilm formation was abolished in a markerless *cdgF* deletion mutant, which contains lower cellular levels of c-di-GMP (Fagerlund et al., 2016). In order to identify gene candidates that are potentially affected by lower levels of c-di-GMP, a whole-genome transcriptional profiling analysis comparing the markerless *cdgF* deletion mutant (Bt407Δ*cdgF*; Table 1) with the isogenic wild-type strain was performed with exponentially growing cells, after 1.5 h of growth in planktonic culture. The microarray analysis revealed that among only three *B. thuringiensis* 407 genes differentially regulated in the *cdgF* deletion mutant during early exponential growth (using cutoffs of two-fold up- or downregulation and false discovery rate (FDR)-adjusted p-value < 0.05; Supplementary Table S2), BTB_RS05575 (GenBank: BTB_c11270) was downregulated 2.5-fold in the *cdgF* deletion mutant, suggesting that this gene was directly or indirectly induced by CdgF. Expression of the two other genes, one encoding a putative aldo/keto reductase and the other a putative MFS-type transporter, was upregulated 2.5-fold (Supplementary Table S2).

### 3.2 Sequence analysis suggests that the protein encoded by BTB_RS05575 (BTB_c11270) is a member of the MSCRAMM family

BTB_RS05575 (BTB_c11270) was interrupted by a gap between two contigs in the first available draft genome sequence of *B. thuringiensis* 407 (GenBank accession number ACMZ00000000) and is annotated as a pseudogene due to a frameshift mutation in the closed *B. thuringiensis* 407 genome (RefSeq: NC_018877.1; (Sheppard et al., 2013)), potentially due to challenges in assembly of repeat regions. We therefore designed PCR primers (Supplementary Table S1) matching the contig ends in ACMZ00000000, PCR amplified across the gap, and sequenced the gap fragment. This approach revealed a novel 192 bp sequence closing the gap between the two contigs, and corrected the frameshift mutation present in the gene sequence in NC_018877.1. The obtained sequence of the closed and intact BTB_RS05575 gene and organization of the surrounding sequence is shown in Supplementary Fig. S1. The protein encoded by BTB_RS05575 (BTB_c11270) in *B. thuringiensis* 407 is an ortholog of BC1060 from *B. cereus* ATCC 14579. Analysis of the BTB_RS05575 (BTB_c11270) promoter region showed that it contains a match to a class I c-di-GMP responsive riboswitch (GEMM), originally identified and named Bc2 in *B. cereus* ATCC 14579 by Sudarsan and co-workers (Sudarsan et al., 2008). This riboswitch was shown to respond to increased levels of c-di-GMP by switching on transcription of the downstream gene (Lee et al., 2010, Tang et al., 2016), suggesting that this is the mechanism by which lower c-di-GMP levels in the *cdgF* deletion mutant mediates regulation of expression of BTB_RS05575 (BTB_c11270)*.*

**Fig. 1:**
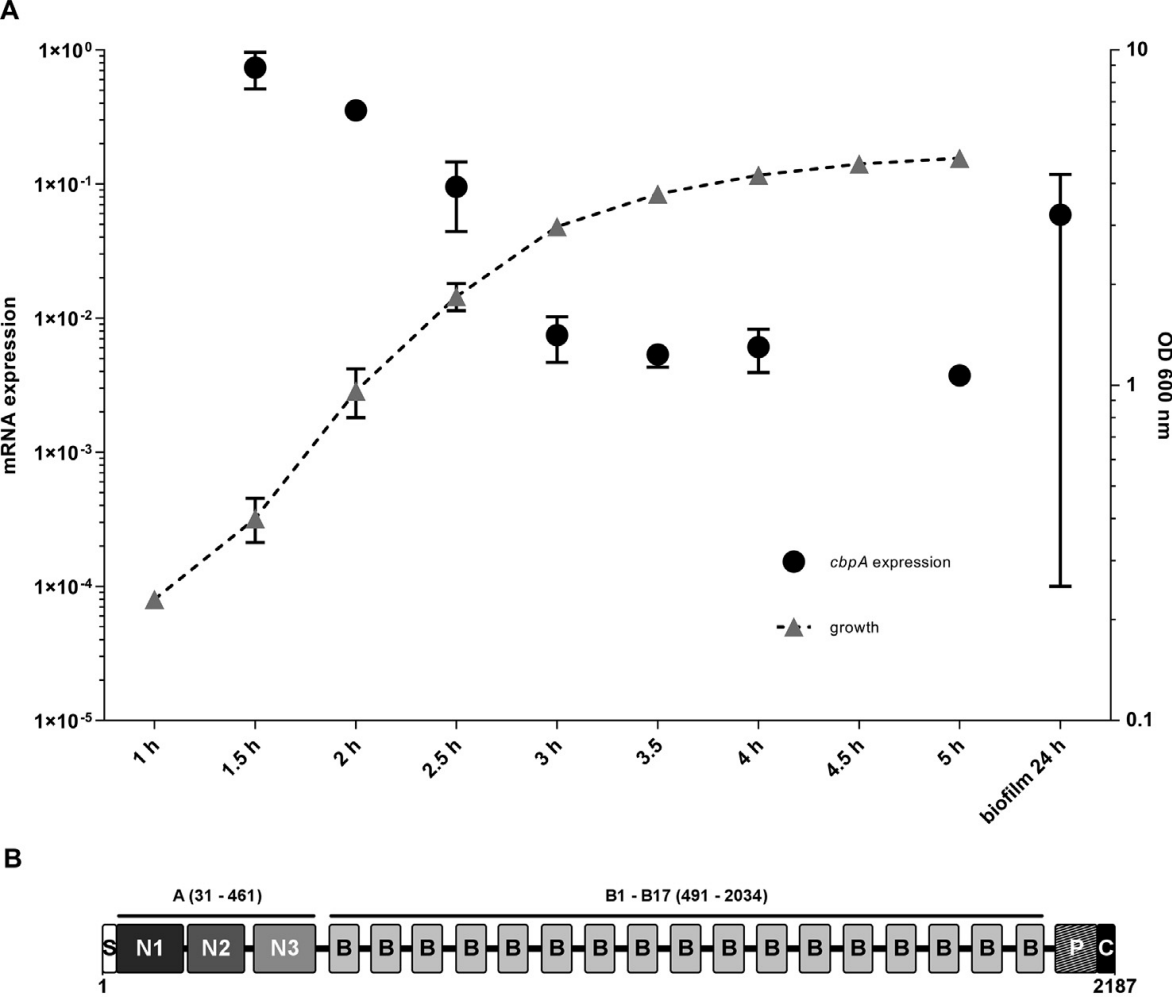
(A) Relative gene expression of *cbpA* during planktonic growth and in 24 h biofilm of *B. thuringiensis* 407, investigated by RT-qPCR. Gene expression of *cbpA* was normalized against the expression of three internal reference genes: *gatB/yqeY*, *rpsU* and *udp*. The growth curve is visualized by a dotted line. The mean and standard deviation of four biological replicates (independent experiments) are shown. (B) Schematic representation of the domain organization of the full-length CbpA protein, showing the signal peptide sequence (S), the predicted subdomains of the A-region (N1, N2, and N3), the Cna_B-type domain repeats of the B-region (B), the proline-rich repeat region (P), and the cell wall sorting signal containing the LPXTG sortase substrate motif (C).

The corrected BTB_RS05575 (BTB_c11270) coding sequence in *B. thuringiensis* 407 is predicted to be 2187 amino acids in length, and contains an N-terminal signal peptide and a C-terminal cell wall sorting signal comprising an LPXTG sortase substrate motif (Gaspar et al., 2005). The processed protein is predicted to have a molecular mass of 236 kDa and a length of 2157 amino acids. Protein domain analysis using InterProScan (Jones et al., 2014) further revealed that BTB_RS05575 (BTB_c11270) shares the typical domain structure of proteins belonging to the group of MSCRAMMs (microbial surface components recognizing adhesive matrix molecules). These proteins are typically characterized by the presence of an A-domain which facilitates binding to extracellular matrix molecules such as collagen, fibrinogen or fibrinogen, the LPXTG cell-wall anchor, and often a B-repeat region (Patti et al., 1994). The non-repetitive N-terminal A-region of BTB_RS05575 (BTB_c11270) was predicted to contain a fibrinogen binding domain (Interpro IPR011252; residues 31–175), a collagen binding domain (Pfam PF05737; residues 184–307), and a fimbrial isopeptide formation D2 domain (Interpro IPR026466; residues 327 to 461) which among others is found in the backbone of the pilus of *Streptococcus pneumoniae* (Spraggon et al., 2010) (Fig. 1B). The two N-proximal domains (aa 31–307) however shared only low primary sequence identity (17–20%) with well-characterized binding domains from other MSCRAMM proteins, such as the N2-N3 domains of fibrinogen-binding SdrG from *Staphylococcus epidermidis* (Ponnuraj et al., 2003), *S. aureus* ClfA (Ganesh et al., 2008) and *S. aureus* SdrD (Wang et al., 2013), and the N1-N2 domains of the collagen-binding protein Cna from *S. aureus* (Zong et al., 2005). In accordance with current terminology the subsection aa 31–307 of the A-region of BTB_RS05575 (BTB_c11270) will be referred to as the N1 and N2 domains, while the subsection covered by residues 327–461 will be referred to as the N3 domain. The B-repeat region in BTB_RS05575 (BTB_c11270), covering residues 491–2034, is composed of 17 units of the Cna_B-type repeat domain (Pfam PF05738). Cna_B repeats were first observed in the B-region of the *S. aureus* collagen binding MSCRAMM protein Cna (Deivanayagam et al., 2000), but are also found in Gram positive pilins (Krishnan, 2015). Also, a proline-rich region composed of eight highly similar repeats with the consensus sequence PGTP[N/D]PEK is located between the B-region and the LPXTG sortase substrate motif (residues 2059–2149). Such proline-rich short repeated sequences are present in the C-terminal region of numerous gram positive cell wall-anchored surface proteins, including *S. aureus* Cna (Symersky et al., 1997). Based on the domain structure of BTB_RS05575 (BTB_c11270), we named the protein CbpA (Collagen binding protein A).

### 3.3 Expression of *cbpA* peaks during early exponential growth

Investigation of *cbpA* mRNA expression levels throughout the *B. thuringiensis* 407 growth phase in a planktonic culture by RT-qPCR revealed an expression peak in the early exponential growth phase, and a substantial expression decline throughout exponential growth (Fig. 1A). Expression of *cbpA* was reduced by 99% after 3 h cultivation compared to its highest expression at 1.5 h, which was the earliest time point of analysis. The level of *cbpA* expression in a 24 h biofilm was roughly the same as in a planktonic culture after 2.5 h incubation.

### 3.4 Distribution of *cbpA* in the *B. cereus* group population structure

**Fig. 2:**
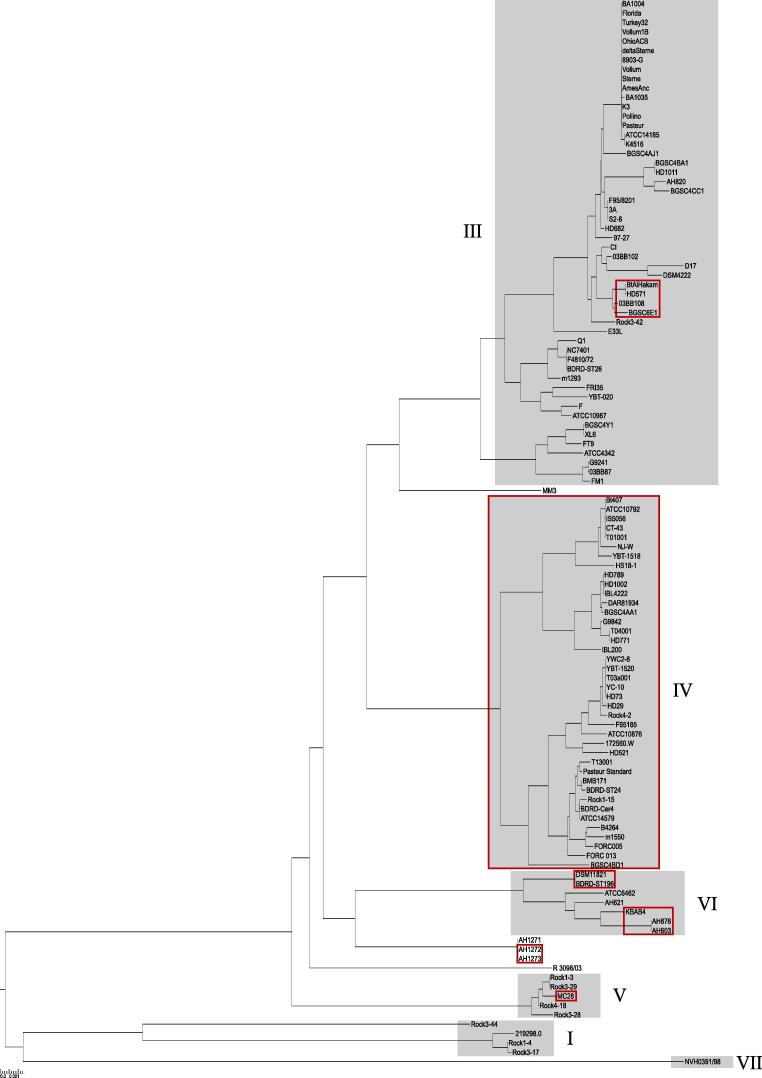
Distribution of *cbpA* within the *B. cereus* group. A phylogenetic tree representative of the *B. cereus* group was constructed based on MLST data, using the distance based Neighbour-Joining method BioNJ. Phylogenetic clusters I and III–VII are indicated. Strains that contain the gene *cbpA* are marked with red boxes. (For interpretation of the references to colour in this figure legend, the reader is referred to the web version of this article.)

Since *cbpA* was under c-di-GMP transcriptional control through its upstream riboswitch (Sudarsan et al., 2008, Tang et al., 2016) and the CdgF diguanylate cyclase and most of the other proteins potentially involved in c-di-GMP metabolism are highly conserved within the *B. cereus* group (Fagerlund et al., 2016), we investigated the phylogenetic distribution of *cbpA*. All strains belonging to the *B. cereus* group for which fully sequenced and closed genomes were available through NCBI at the time of analysis were checked for the presence of *cbpA* by using BlastN and BlastP, using *cbpA* and CbpA sequences from *B. thuringiensis* 407 as query. Interestingly, all strains belonging to the phylogenetic cluster IV carry the *cbpA* gene, in addition to a few other strains within phylogenetic groups III, V and VI (Fig. 2). The surrounding chromosomal region was investigated for evidence of horizontal gene transfer (HGT), but organisation of the surrounding genes in the strains included in the study (Fig. 2) and the fact that the GC content of this area conforms with the average GC content of the rest of the chromosome of strain *B. thuringiensis* 407, does not suggest that *cbpA* was acquired by HGT. Furthermore, no evidence of transposable elements was identified in this region by running the chromosome of the *B. thuringiensis* 407 strain through the ISsaga2 semi-automatic pipeline (Varani et al., 2011) and no indication for the presence of prophages in this region was found by using the phage identification tool PHASTER (Arndt et al., 2016). Phylogenetic cluster IV is comprised of *B. cereus* and *B. thuringiensis* strains which are often isolated from non-clinical sources including nature environments and insects (Guinebretiere et al., 2008), http://mlstoslo.uio.no). *cbpA* was not identified in *B. anthracis* or any emetic-toxin producing strains which group within phylogenetic cluster III, and only four of the 40 *B. cereus* or B. *thuringiensis* strains belonging to cluster III contained *cbpA*.

The highest variability within CbpA of the analysed strains was observed in the B-region and in the proline-rich region (Supplementary Fig. S2), while the N1-N3 putative binding domains were highly conserved, generally at >90% identity. Notably, several of the analyzed strains were found to contain the *cbpA* gene, but based on the genome sequences present in GenBank carry genetic changes implying the strains may carry *cbpA* pseudogenes or alternatively, as found in our resequencing of *B. thuringiensis* 407 *cbpA*, may be the result of genome sequencing errors due to extensive internal repeat regions in the *cbpA* gene. All strains that contained the *cbpA* gene (or pseudogene) also contained a predicted class I c-di-GMP binding riboswitch in the upstream untranslated region (UTR).

### 3.5 CbpA of *B. thuringiensis* 407 binds collagen during early exponential growth

**Fig. 3:**
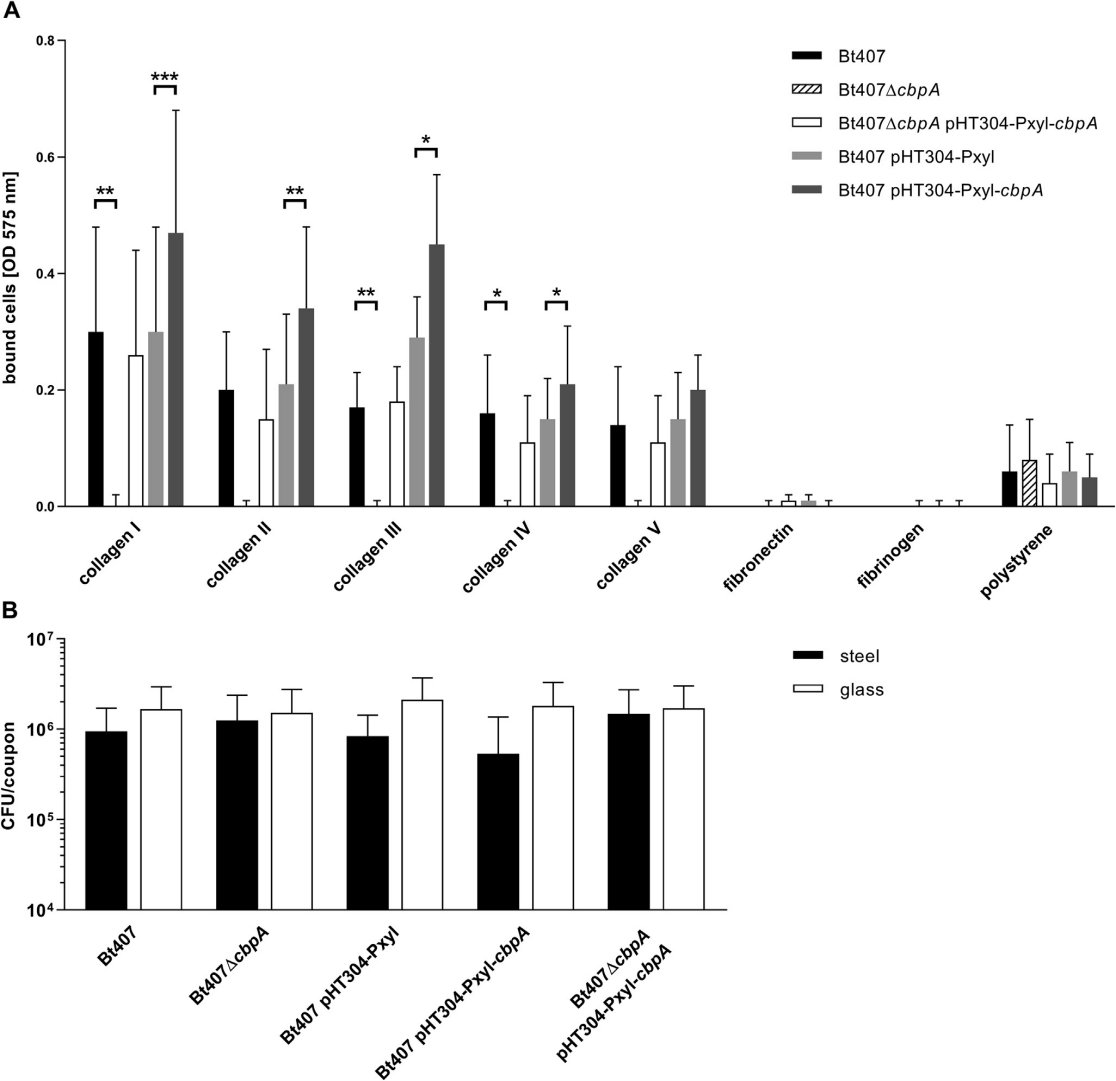
Effect of CbpA on adhesion in *B. thuringiensis* 407. Binding of *B. thuringiensis* 407 wild type (Bt407), *cbpA* deletion mutant (Bt407Δ*cbpA*), empty vector control strain (Bt407 pHT304-Pxyl), CbpA overexpression strain (Bt407 pHT304-Pxyl-*cbpA*) and complementation strain (Bt407Δ*cbpA* pHT304-Pxyl-*cbpA*) to extracellular matrix molecules and abiotic surfaces was investigated by a bacterial whole cell adhesion assay. (A) Bacterial cells bound to collagen, fibronectin and fibrinogen coated onto polystyrene plates were visualized and quantified by staining with methyl violet 6B. The mean and standard deviation of three independent experiments are shown for each extracellular matrix molecule tested, except for the binding assays against collagen I and collagen IV where eight and four independent experiments were performed, respectively. (B) Adhesion of cells to steel coupons and glass plates was measured by CFU count. The mean and standard deviation of three independent experiments for each abiotic surface are shown. For both experiments (A, B) a two-tailed paired *t*-test was performed to test for statistical significance, comparing the deletion mutant and the complementation strain with the wild type, and the overexpression strain with the vector control strain, respectively, for each of the tested conditions (surfaces) separately (*P < 0.05; **P < 0.01; ***P < 0.001).

To uncover a possible host cell molecule binding to CbpA, a markerless gene deletion mutant Δ*cbpA* and corresponding *cbpA* complementation- and overexpression strains utilizing the xylose-inducible vector pHT304-Pxyl were constructed (Table 1). The capability of these strains to bind to different adhesive host cell matrix molecules was examined with a bacterial whole cell adhesion assay. Considering that *cbpA* reached its peak expression during the early exponential growth phase (Fig. 1A) bacteria were harvested at this point of growth for the assay, and adhesion was tested for collagen types I – V. For the *B. thuringiensis* 407 Δ*cbpA* deletion mutant (Bt407Δ*cbpA*), binding was completely abolished to all types of collagen tested, while the *cbpA* overexpression strain (Bt407 pHT304-Pxyl-*cbpA*) showed statistically significant increased adhesion to collagens I – IV compared to the vector control strain (Bt407 pHT304-Pxyl) (Fig. 3A). As a control, adhesion was restored in the complementation strain (Bt407Δ*cbpA* pHT304-Pxyl-*cbpA*) to roughly the same levels as in the wild type strain for all tested collagens (no statistically significant difference). The adhesion assay clearly revealed that under the tested conditions, CbpA is essential for binding to collagen.

As described above, prediction protein domain analysis methods potentially suggested additional binding ligands for CbpA, and specific MSCRAMM proteins are known to have the ability to adhere to other extracellular matrix molecules than collagen (Wann et al., 2000, Vazquez et al., 2011, Larson et al., 2013). Deletion of *cbpA* did however not produce any difference in adhesion to either fibrinogen or fibronectin compared to *B. thuringiensis* 407 wild type. In fact, under the tested conditions none of the strains produced detectable binding of the bacterial cells to wells coated with either of these two molecules (Fig. 3A).

The influence of CbpA on *B. thuringiensis* 407 adhesion to abiotic surfaces was also investigated. The control from the binding assay for biotic factors included a background test, showing that CbpA did not affect bacterial whole cell binding to polystyrene (Fig. 3A). Binding to steel and glass surfaces was investigated with an assay based on CFU count as described by Hayrapetyan and co-workers (Hayrapetyan et al., 2015). Based on the results of this assay CbpA does not influence either binding to steel or glass in *B. thuringiensis* 407 (Fig. 3B).

### 3.6 Other phenotypes affected by CbpA

**Fig. 4:**
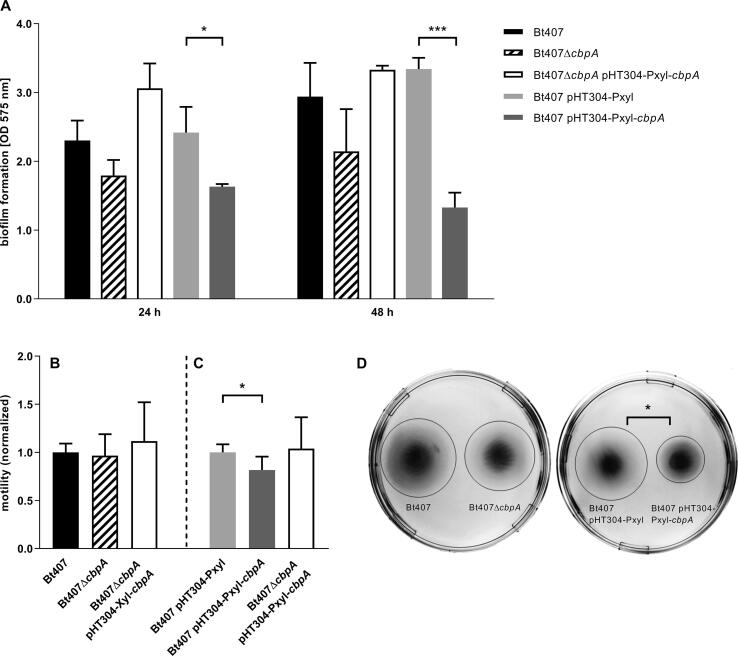
Influence of CbpA on biofilm formation and motility. The *B. thuringiensis* 407 wild type (Bt407), *cbpA* deletion mutant (Bt407Δ*cbpA*), empty vector control strain (Bt407 pHT304-Pxyl), CbpA overexpression strain (Bt407 pHT304-Pxyl-*cbpA*) and complementation strain (Bt407Δ*cbpA* pHT304-Pxyl-*cbpA*) were assessed for biofilm formation and motility. (A) The capability to form biofilm was investigated with an assay based on staining of the biofilm with methyl violet 6B. The mean and standard deviation of three independent experiments are shown. A two-tailed *t*-test was performed to test for statistical significance, comparing the deletion mutant and the complementation strain (respectively) to the wild type, and the overexpression strain to the vector control strain (*P < 0.05; ***P < 0.001). (B-C) Swimming motility was examined after growth for 7 h on LB plates containing 0.3% agar. Shown are the mean values and standard deviation of normalized distances from at least three independent experiments. Within each experiment the *cbpA* deletion mutant was normalized against the wild type strain (B), the CbpA overexpression strain normalized against the empty vector control strain (C) and the complementation strain was normalized against both the wild type and the vector control strain (B, C). A single star (*) symbolises p < 0,05 in a two-tailed paired *t*-test. (D) shows a motility assay plate which displays representative results for the experiment. The swimming zones are marked with circles for better visualization.

Both biofilm formation and motility are regulated by intracellular c-di-GMP levels in many bacteria, including those in the *B. cereus* group (Fagerlund et al., 2016). Adhesion is the first step of the biofilm formation process, and expression of the CbpA adhesin was shown to be positively controlled by a c-di-GMP binding riboswitch (Lee et al., 2010, Tang et al., 2016). Therefore a role of CbpA in both of these cellular processes was investigated. The CpbA overexpression strain (Bt407 pHT304-Pxyl-*cbpA*) formed less biofilm compared to the vector control strain (Fig. 4A), however we did not observe any significant effect on biofilm formation following deletion of *cbpA*.

As for the biofilm assay, we did not observe any change in motility for the *cbpA* deletion mutant compared to its isogenic wild type strain (tested at OD_600_ = 1; Fig. 4B). However, the overexpression strain (Bt407 pHT304-Pxyl-*cbpA)* had a significantly smaller swimming zone compared to the vector control strain (Bt407 pHT304-Pxyl), indicating an impairment in motility upon *cbpA* overexpression (Fig. 4C and D). In order to ensure that the influence of CbpA on motility is not dependent on growth phase, the test was also done with cells harvested during the early exponential growth phase (OD_600_ = 0.2) and stationary phase, obtaining similar results compared to the original assay (Supplementary Fig. S3).

**Fig. 5:**
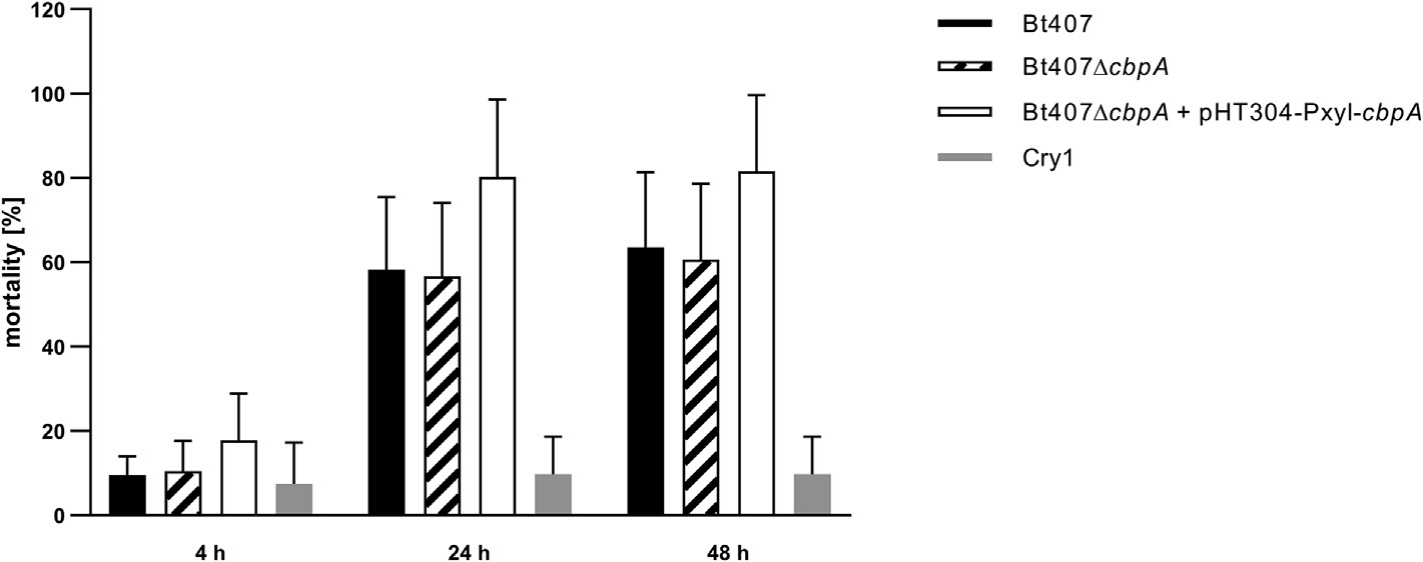
Effect of *cbpA* deletion on *B. thuringiensis* 407 virulence in a *G. mellonella* larvae infection model. Larvae were infected by force feeding with vegetative bacteria of *B. thuringiensis* 407 wild type (Bt407), the *cbpA* deletion mutant (Bt407Δ*cbpA*)*,* the *cbpA* complementation strain (Bt407Δ*cbpA* pHT304-Pxyl-*cbpA*) or Cry toxin alone. The mortality of larvae was recorded in at least four independent experiments. The mean and standard deviation values of all independent experiments are shown.

Since adhesion to host factors is the first step of infection, the function of MSCRAMMs is usually predicted to be connected to infection processes (Patti et al., 1994). In order to investigate if the presence of CbpA on the surface of *B. thuringiensis* 407 cells might play a role during infection, a well-established *in vivo* infection model was utilised, employing *G. mellonella* larvae (Bouillaut et al., 2005, Ramarao et al., 2012, Voros et al., 2014, Kamar et al., 2017). We did not observe any significant phenotype for the Δ*cbpA* deletion mutant compared to the *B. thuringiensis* 407 wild type strain during infection of *G. mellonella* (two-tailed students *t*-test; Fig. 5). There was however a trend towards mortality being slightly but not statistically significantly higher in larvae infected with the complementation strain (*B. thuringiensis* 407 Δ*cbpA* pHT304-Pxyl-*cbpA*) compared to the wild type.

## 4 Discussion

To the best of our knowledge this study presents the first experimental evidence that the *B. thuringiensis* MSCRAMM protein CbpA mediates binding to collagen, an extracellular host cell matrix protein. The CbpA ortholog in the *B. thuringiensis* strain BMB171 has been subject of a study by Tang and colleagues (Tang et al., 2016), which focused mainly on the function of the upstream c-di-GMP riboswitch but also aimed to elucidate the function of this protein in cellular processes. Although Tang and colleagues named this protein Cap (“collagen-adhesion protein”) their previous study does not provide any investigation into an actual binding ligand for this putative adhesion protein, and the name was given solely on the basis of comparison of predicted protein domains, and comparison to proteins with very low sequence homology. As described here (3.2), domain analyses of the primary amino acid sequence predicted both collagen and fibrinogen as possible binding ligands for CbpA/Cap. By utilizing a bacterial whole cell adhesion assay we were able to determine that CbpA is indeed capable of binding to collagen, as adhesion to this extracellular host cell matrix protein was completely abolished in a Δ*cbpA* deletion mutant. In our study, CbpA is essential for *B. thuringiensis* 407 attachment to collagens I, II, III, IV and V, for bacterial cells in exponential growth phase. Results from the adhesion assays further suggest that CbpA is not able to bind to neither fibrinogen nor fibronectin, contrary to several other MCSRAMM proteins (Wann et al., 2000, Vazquez et al., 2011, Larson et al., 2013). We propose that the name of this protein is changed from Cap to CbpA (collagen-binding protein A), in line with existing genetic nomenclature. The name Cap used for an adhesion protein also leads to unnecessary confusion, since within the *B. cereus* group *cap* genes are a hallmark for genes responsible for synthesis of the poly-γ-D-glutamic acid capsule (Cachat et al., 2008), typically found in highly virulent strains.

Members of the *B. cereus* group continue to pose a significant problem in the food industry, and the ability of these bacteria to bind to abiotic surfaces, especially steel which is commonly used in food processing plants, has been investigated previously (Wijman et al., 2007, Hayrapetyan et al., 2015, Galie et al., 2018). Although there are examples of other MSCRAMM proteins having a functional role in attachment of bacterial cells to polystyrene (Shimoji et al., 2003, Schroeder et al., 2009), as well as instances of other cell surface protein structures such as pili playing a role in adhesion of Gram-positive bacteria to abiotic surfaces (Pratt and Kolter, 1998, Giltner et al., 2006), there is no evidence suggesting that CbpA facilitates attachment to surfaces such as polystyrene, glass or stainless steel, substantiating the possibility that CbpA-mediated adhesion is specific to collagen.

Biofilm formation and motility are cellular processes which are typically affected by c-di-GMP signalling (Fagerlund et al., 2016, Jenal et al., 2017). Since *cbpA* expression is regulated at least partly by an upstream c-di-GMP binding riboswitch (Lee et al., 2010, Tang et al., 2016), we aimed to determine if CbpA had a role in mediating these processes. Considering that it has been shown that higher levels of c-di-GMP promotes biofilm formation in *B. thuringiensis* 407 (Fagerlund et al., 2016) and that expression of *cbpA* is positively regulated by c-di-GMP, it could be expected that *cbpA* expression in biofilm cells would be high (Lee et al., 2010, Fagerlund et al., 2016, Tang et al., 2016). Analysis by RT-qPCR however revealed that *cbpA* expression was not at its peak in biofilm cells, but in planktonic cells during the early exponential growth phase, possibly indicating that expression of *cbpA* is subject not only to transcriptional control by c-di-GMP, but potentially also by other mechanisms. We did not detect a significant difference in biofilm mass nor in motility upon *cbpA* deletion in *B. thuringiensis* 407. In contrast, Tang and colleagues (Tang et al., 2016) report both increased biofilm formation and higher motility in a *cbpA* deletion mutant of *B. thuringiensis* BMB171. This is surprising, since *B. thuringiensis* 407 and *B. thuringiensis* BMB171 are closely related strains within the *B. cereus* group, and the sequence identity between the predicted binding domains N1-N3 of CbpA in these two strains is 97%. Although we could not relate the absence of CbpA from the *B. thuringiensis* 407 cell surface to biofilm or motility phenotypes, the overexpression of CbpA in *B. thuringiensis* 407 led to a decrease in both biofilm formation and motility. This is in contrast to the common pattern of c-di-GMP regulatory networks where motility and biofilm formation are normally oppositely regulated (Jenal et al., 2017), and which is also found within the c-di-GMP regulatory network in *B. thuringiensis* 407 (Fagerlund et al., 2016), possibly suggesting that the observed effects of CbpA overexpression on biofilm formation and motility may be caused by simple physical interruption of biofilm and motility processes due to unnaturally high amounts of CbpA protein on the cell surface, rather than representing a true biological function of CbpA. CbpA is a large protein with a predicted molecular weight of 236 kD and contains a long repetitive B-region which is thought to help project the binding domain A away from the bacterial cell surface, presenting it to its ligand(s) for binding (Rich et al., 1998, Deivanayagam et al., 2000, Jemima Beulin and Ponnuraj, 2017). With the predicted size and structure of the CbpA protein one may speculate that increased amounts of the protein present at the cell surface could possibly disrupt normal movement of the flagella, as well as potentially disturb the accessibility of other surface adhesins, leading to decreased swimming motility and a decreased ability of the bacteria to attach to surfaces and/or to each other.

Most infections caused by *B. cereus* are gastrointestinal tract infections, but the bacterium is also known to cause a variety of other infections, particularly in immunocompromised patients (Drobniewski, 1993, Bottone, 2010). Collagen is the major component of connective tissue and the extracellular matrix in mammals, and is found in all organs including the gastrointestinal tract. The collagen content in the human intestine was found to be predominately collagen I (68%), collagen III (20%) and collagen V (12%) (Graham et al., 1988), and collagen is located in the submucosa of the gastrointestinal wall, but also in the basement membrane which is attached to the overlaying epithelium and contains collagen IV (Visser et al., 1993, Beaulieu et al., 1994). Collagen is also found in invertebrates including insects, where it is located under the epidermis and around organs including the midgut, and there is evidence which supports that insect collagens are similar to mammalian collagen I and IV (Francois et al., 1980, Francois, 1985, Lunstrum et al., 1988). The phylogenetic distribution of CbpA with its predominance in phylogenetic group IV may imply that this protein could be more abundant in *B. cereus* group strains isolated from non-clinical sources. Interestingly however, all collagens that were used in the adhesion assay in this study originate from mammalian tissues, providing a potential for CbpA to make a contribution to attachment of *B. cereus* group strains to tissues also during human infection. Also, while *B. thuringiensis* is often considered non-pathogenic for humans and is widely used as an biological pesticide (Jouzani et al., 2017), some studies have suggested that the bacterium may have pathogenic potential to humans (Damgaard et al., 1997, Hernandez et al., 1998, Helgason et al., 2000) and *B. thuringiensis* strains generally contain the same chromosomal virulence factors that are often associated with human infections caused by *B. cereus*. This includes the Hbl, Nhe and CytK cytotoxins (Ngamwongsatit et al., 2008, Guinebretiere et al., 2010), the function of which during *B. cereus* group infections is not completely elucidated. Yet these proteins are generally theorized to cause food-borne diarrhoeal disease by disruption of the epithelial layer, functioning as enterotoxins. Also, the insecticidal Cry toxins of *B. thuringiensis* function by forming pores in the epithelial cells in the insect midgut (Nielsen-LeRoux et al., 2012). One may therefore speculate that expression of collagen-binding proteins like CbpA could potentially facilitate attachment to any exposed collagen after disruption of the epithelial layer, and thereby assist in the infection process in different hosts. The *in vivo* infection assay in the wax moth *G. mellonella* larvae did not reveal a significant decrease in mortality for the *cbpA* deletion strain. Quite possibly, this could be explained by genetic redundancy, as *B. thuringiensis* 407 contains two other proteins that are annotated as collagen adhesins, as well as an ortholog to *B. anthracis* BA0871 for which binding of collagen has been shown experimentally (Supplementary Table S3) (Xu et al., 2004). Although our results suggest that CbpA is not essential for infection of insects by *B. thuringiensis,* the protein could still facilitate adhesion during the colonisation of a host and it is interesting to observe that a slight trend towards increased virulence was observed when the *cbpA* gene is expressed from the low copy plasmid pHT304-Pxyl in the complementation strain. Also, *B. thuringiensis* has been suggested to have the capacity to form biofilm in the insect gut (reviewed in Majed et al., 2016). In the previous work by Tang and co-workers (2016) it was reported that the CbpA ortholog (named Cap) from *B. thuringiensis* strain BMB171, although carrying a similar complement of putative collagen adhesion proteins to *B. thuringiensis* 407, indeed affected virulence towards the insect larvae *Helicoverpa armige*ra. Here the *in vivo* assay was however run under substantially different experimental conditions, using young larvae and bacteria in the sporulation stage, and the effect measured over  six days following free feeding. This is different from the *G. mellonella* analysis performed in the present study, with a one shot dose of a controlled number of vegetative bacteria on large larvae, possibly explaining the difference in obvious impact.

This study presents the first experimental proof that the extracellular matrix protein collagen is a ligand of the CbpA cell surface adhesin, and that CbpA-mediated adhesion is collagen-specific and not implicated in adhesion to fibrinogen or fibronectin, nor to tested abiotic surfaces. CbpA is present in a range of *B. cereus* group strains, and may constitute a novel surface protein potentially involved in adhesion processes during infection.

## Declaration of Competing Interest

The authors declare that they have no known competing financial interests or personal relationships that could have appeared to influence the work reported in this paper.
