# Supplementary material for: Bacillus thuringiensis CbpA is a collagen binding cell surface protein under c-di-GMP control
Source: Cell Surf. 2019 Aug 23;5:100032. doi: 10.1016/j.tcsw.2019.100032 (PMC7423583; doi:10.1016/j.tcsw.2019.100032)
Supplement: Supplementary data 1 [file mmc1.docx]

**Supplementary Data**

***Bacillus thuringiensis* CbpA is a collagen binding cell surface protein under c-di-GMP control**

Sarah Finke^1^, Annette Fagerlund^1,¶^, Veronika Smith^1^, Veronica Krogstad^1^, Mimmi Jingxi Zhang^1^, Athanasios Saragliadis^2^, Dirk Linke^2^, Christina Nielsen-LeRoux^3^, Ole Andreas Økstad^1,*^

*^1^Centre for Integrative Microbial Evolution and Section for Pharmaceutical Biosciences, Department of Pharmacy, University of Oslo, Norway, ^2^Department of Biosciences, University of Oslo, Norway, ^3^INRA UMR1319 Micalis AgroParisTech, Paris Saclay University, Jouy-en-Josas, France.*

^¶^ Present address: Nofima AS, Postboks 210, NO-1431 Ås, Norway

* Corresponding author at: Department of Pharmacy, University of Oslo, PB 1068 Blindern, 0371 Blindern, Norway.

*Email address:* [aloechen@farmasi.uio.no](mailto:aloechen@farmasi.uio.no) (O. A. Økstad)

**Supplementary Table S1**. Primers used in this study. Inserted restriction sites are underlined.

| Primer name | Sequence (5’ to 3’) | Function |
| --- | --- | --- |
| 02561-F2 | AAAGGTGGGAAGGCAAAAAT | PCR and sequencing Bt407 *cbpA* between contig gaps |
| 02561-R2 | TACATACGGGAAACGGGAAG |  |
| Bt407orf02561upF | ACGCAGAGAAGAAAGGGAGA | Cloning region upstream of *cbpA* for pMAD (*Mfe*I) |
| Bt407orf02561upR | TTCAATTGCATAGTTCCCTCCTATT |  |
| Bt407orf02561downF | CAGCAATTGTAATAAAAGTGAAAAGGTATCC | Cloning region downstream of *cbpA* for pMAD (*Mfe*I) |
| Bt407orf02561downR | CATTCACCGTACGCATCATC |  |
| Up-02561-F | GTGCAATGTAAAGGGGTTGC | Confirming *cbpA* deletion |
| Down-02561-R | GTCTAACTTTCCCGCCATCA |  |
| BC1060-F-XbaI | CATTCTAGATGCAACCGGACGGTCTTTT | Cloning of *cbpA* into pHT304-Pxyl |
| BC1060-R-KpnI | TTAGGTACCGGAAACGGGAAGAATTTTG |  |
| 02561-BC1060-F3 | ATGTAACAGGGGACGGCATA | RT-qPCR |
| 02561-BC1060-R3 | TACTGCCCCGTTTTCTTTGT | RT-qPCR |

**Supplementary Table S2**. Global expression profiling (microarray) results for the markerless *B. thuringiensis* 407 Δ*cdgF* deletion mutant relative to *B. thuringiensis* 407 wild type (exponential growth phase), showing genes with a fold change > 2 and False Discovery Rate (FDR) adjusted p-value < 0.05.

| ***B. thuringiensis* 407 ^a^** | ***B. cereus* ATCC 14579 ^b^** | **Predicted function** | **log_2_FC** | **Fold**  **change** | **p-value (FDR-adjusted)** |
| --- | --- | --- | --- | --- | --- |
| Genes downregulated in the Δ*cdF* deletion mutant (putatively induced by the activity of CdgF) | | | | | |
| BTB_c11270/  BTB_RS05575 | BC1060 | Putative collagen adhesion protein | -1.36 | 2.57 | 7.4E-10 |
| Genes upregulated in the Δ*cdF* deletion mutant (putatively repressed by the activity of CdgF) | | | | | |
| BTB_c34520/ BTB_RS16935 | BC3392 | Aldo/keto reductase YtbE | 1.36 | 2.56 | 1.2E-6 |
| BTB_c52900/ BTB_RS25990 | BC5058 | MFS-type transporter | 1.31 | 2.47 | 2.3E-9 |

^a^ Locus tags are taken from GenBank entry CP003889.1 and NCBI RefSeq entry NC_018877.1, respectively.

^b^ Ortholog (gene which microarray oligo matches) in *B. cereus* ATCC 14579.

**Supplementary Table S3**. Putative collagen adhesion proteins in *B. thuringiensis* 407, in addition to CbpA.

| Protein ID | Name/description | Locus tag in NC_018877.1 | Length | Description including protein motif recognition prediction with InterProScan |
| --- | --- | --- | --- | --- |
| WP_001232041.1/  AFV16652.1 | Collagen-bindiing protein | BTB_RS04755  BTB_c09570 | 1086 | Shares 71% sequence identity  to BA0871  Binding of collagen was experimentally shown for BA0871 in *B. anthracis* (Xu, Liang, Chen, Koehler, & Hook, 2004)  1 x Choice-of-anchor A domain (IPR0265889)  5 x CNA_B-type repeat domain (Pfam PF05738)  Annotated as partial in NC_018871.1: Gram-Positive LPXTG-containing cell wall anchor domain (InterPro IPR019948) is not complete |
| WP_000520161.1 | Annotated as  collagen-binding protein | BTB_RS17655 | 1093 | 1 x TQXA-containing domain of unknown function, is found in some adhesins (InterPro IPR023849)  4 x CNA_B-type repeat domain (Pfam PF05738)  4 x T-Q ester bond containing domain  (PFAM 18202)  1 x Gram-Positive LPXTG-containing cell wall anchor (InterPro IPR019948) |
| WP_000732739.1/  [WP_003272463.1](https://www.ncbi.nlm.nih.gov/protein/489365611) | Annotated as  collagen-binding protein | BTB_RS27340 | 3236 | 2 x Fibrogen-binding domain 1 (Interpro IPR011252)  5 x collagen binding domain (InterPro [IPR008456](http://www.ebi.ac.uk/interpro/entry/IPR008456))  21 x CNA_B-type repeat domain (Pfam PF05738)  1 x Gram-Positive LPXTG-containing cell wall anchor (InterPro IPR019948)  Predicted binding Domain N1 of CbpA shows 30 % pairwise identity to a 93 aa region within 2nd predicted Fibrogen-binding domain in this protein  Predicted binding domain N2 of CbpA shows   - 28 % pairwise identity to a 108 aa region within 1^st^ predicted collagen binding domain - 27 % pairwise identity to the 2^nd^ predicted collagen binding domain |


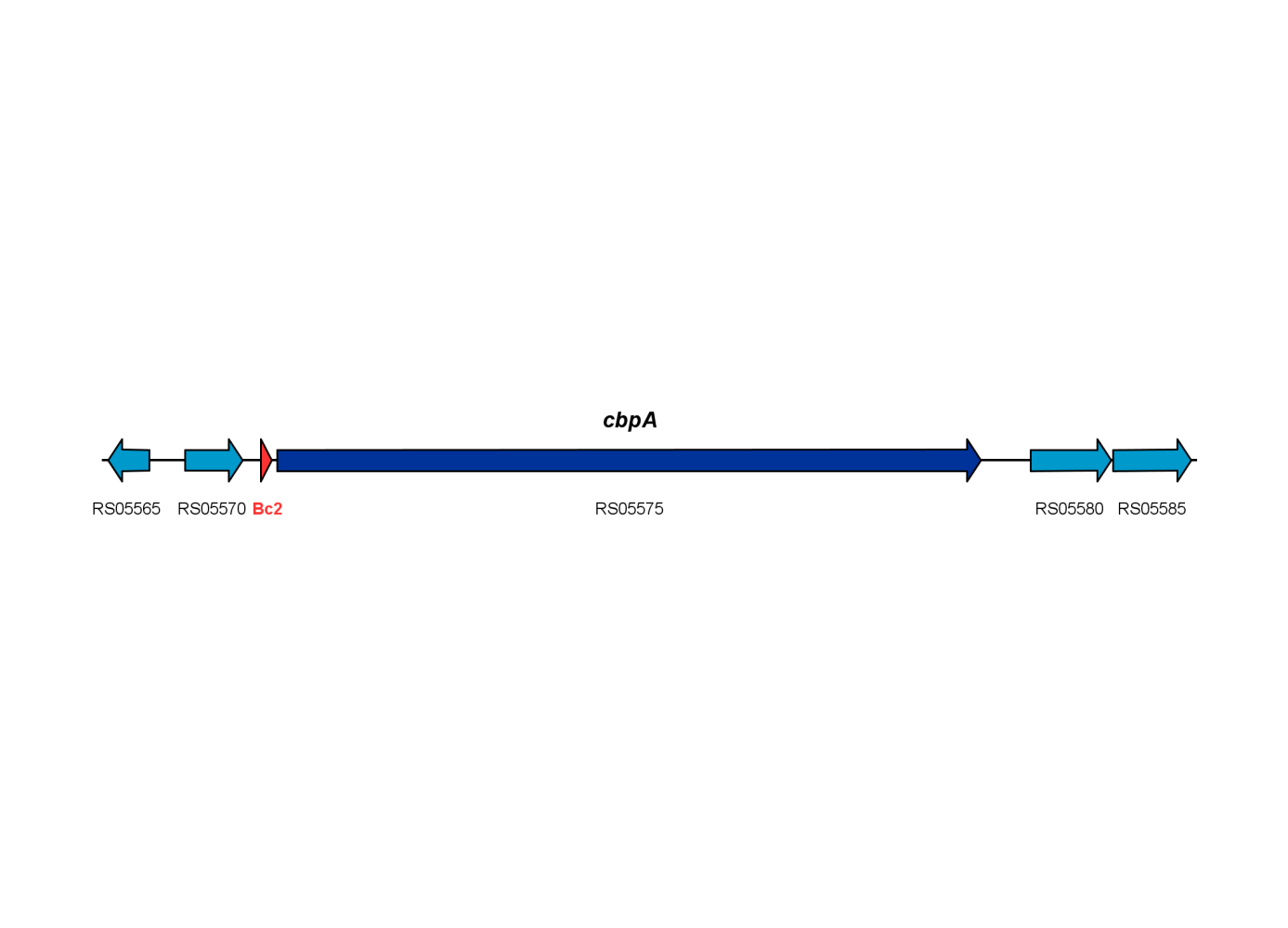


**B**

**A**


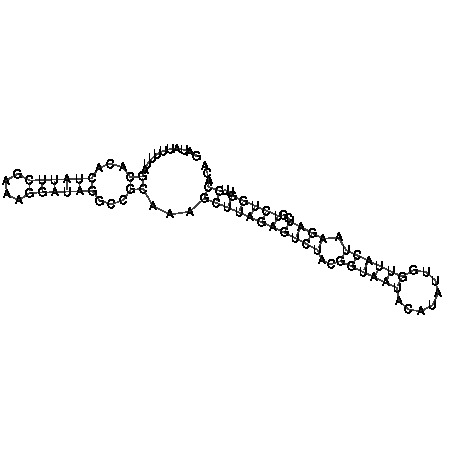
ATATACGAACTATATAATGCGAAAATAATTCACCATTGCTTCTTTAATGCAGTCGTGAATTATTTTTTTTATTAGAAAAATAGAACAATTTTATAGAGTATTGAATTATAACCGTCCAGACGGTATAGTAATATTTGGTTTAAGAGGAAAATGAAACAAAAAAAAGAATAAAAGATATTTTTAGCACACTATTCGAAAGGATAGGCCGCAAAGCTTAGAGTCTACGGTAATACATATTGGTTACTAAGATCGTCTGGTTGCACATTTTTGATGCAACCGGACGGTCTTTTTTTGTTATATAAATATAAAAAATAGGAGGGAACT**ATG**

**C**

ATGCAAATGAAAAGTACTAAAAGACATATTTCAATGGTTTCATTAGTTTTTCTAGTATTATTTTCAGTGCTGAATGTGTGGGCTCCAGTTATTCAAGCTGCTGTGATGAAAAGTCCAGTGGACGAAATTAATATTTCCCGTACTGATGGCACGACATCTGAACCGTATCAAGCCTCAGATGGTATGAAAGTAGAAGTGAAGTGGTCAGCTAAAGAAAAAATAAAAAGTGGAGATCAATTCACAATTGATATGCCAAAAGAGTTTCGAAAAGACCTTATGAATATGAGTTTTCCTTTAAAAGATGCTGAAGGAAAAACAGTTGGTACATGTGAGATGAAAAAGGGTCTATTAACGTGTACTATGGGTAATTACGTAGAAGGAAAGAATAACATTAAAGGTTCTTTATTCGTAGAATTCTACTTTGGTTTAGAAGCATATGATGGTGTTAAAGAAATTCCATTAGAATTTAACGTTGATGGTCAAATCGTTAATAAAGAAGTAAATGTAAGTAATACGACAGAGAGACCGAAGCCACAGCCCAATACAGATAATCTATTGAAATGGGGTTCTTATAATCAAGAAGATCCTTCGATTGCTGATTGGCTTGTATATGTAAATGCAACTGGAACAGAAATGCAAGATCTTAAATTAACAGATACATTAGGACCTGGGCATGAGTTAATTACAGACAGCGTAGTATTAGAAGAGGCTGTATTTGAAGATGGTTATGCACCAACAAATATTAAGCCAGCAGATTTATCTAACATTAAAATAAATGCAACGAAGACTGGATTTACAATTGAGTTTCCAGACAGTTCAAAAGGCTATATTTTAAGATATAAAACAAAGGTTACAAATCCTGCTGCGAAGCCTCATAAAAATACTGTGAAATTAGAAGGTAAAAATATTAAAACTGAAGAAAAAGTAGGACAAGTATTTGTAAGTGGTGGGGGAGGATCCGGTTCAGGTGACAATAATCCACCTAGCATTGAAAAAAATATAGTTGATGAGAATGGCAAGCTTGTAGAGAATGAGCAGTTAACACAAATGGATCAAGTTATTCAATACCAAGTTGGTACACATATACCGAACGATCCTCCTAAATACACTTCTATGGTAATTAGTGATGATTTAGAAGATGTTTTAGAAGTATTAGAGGCAAAAGTGTATGATCAAAATGGCCAAGATATTACTTCTAAAGGAACGTTAAATATAGATAAACAAAGAAGTGAAGTAACATTTACGTTTGGTGAGAGTTTTGACTATAAGTCATATGAAGACCAGATAATTAACCTTAGTATTAAGGCGAAAATCAAAAATAATGCAGACTTATCTTCTTACGTAGATAAGAAGATTCCTAATAAAGCGGAATTACATTTTGATGATAAAACTTTAACATCAAAAGAAGTAACTATTACGCCGCCTGAAGCTCCAAAAGATGGTACAGTATCGCTTCATAAAATAGATTCTGAGAATCCGAACAAAGGGTTAAAAGCTGCCGAATTTGAAGTCCGAAATAGTGCAAATGAAGTTGTTGCAAAACTGAAAACGGACGAAAAAGGTTTTTCAGTACCTCAAACTTTAGCTCCTGGGACGTATAAAGTATATGAAACAGTAGCACCAGAAGGATATCAAAAATTAACAAGTCCAGTAGAGGTTACACTTCAAGCTGGAGAAACAAAAACAATTGAAATTAAAAATACTATGCAAAAGGGTCAAATTGAAGTAAAGAAAATTGATTCGGAAAATGGTGAGAAACCATTAGCAAATGCAGAGTTTGATATAGTAAAAGACGGTGTAGTAGTCGAACATATTGTTACAGATAAAGATGGTAAGGCAATCTCTAAACCGTTAGCACCAGGAAAATATATTTTAAAAGAAACAAAAGCACCTGAAGGCTACCAATTAAAAGAAACAGAGTTCGAAGTAAATGTAACAGGGGACGGCATATTCCCAATACAAGTGGAAAATGCAATGGTAGACAAAGGTAATATAGAAATTACAAAAGTGGACAAAGAAAACGGGGCAGTATTAGCCGGTGTCGAATTTGAAGTCCAAGATGAGAAAGATGGCGTAGTAAGAAAAGTAGTAACAGATAAAGAAGGAAAAGCAAACGTTTCAGATCTATCAGTAGGAAAGTACAAATTAGTAGAGACGAAAAGCTTACCAGGTTACAAAAATCTAGCAGAACCAGTACTATTTGAAATTACAAAAGGTATGACAAAAGCTTTAGTAATAAAAGTAGAAAATGAGCAGTTAGACAAAGGCTCAGTAGAAATTACAAAAATAGATAAAGATAGTCAAAAAGTATTAGAAGGCGTAGTCTTCGAAGTGCAAGATGAGCAAGGAAAAGTAGTAACAGAAGTAAAAACAGATAAAGATGGTAAAGCAAAAATCTCAGATTTATCAGTAGGAAAGTACAAATTAGTAGAGACGAAAAGCTTACCTGGCTACAAAAAGCTAACAGAACCAGTATCATTCGAAATTACAAAAGGTATGACAACAGTCCTATCATTGAAAGTAGAAAATGAACAGTTAGATAAAGGTTCAGTAGAAATTACAAAAGTAGATAAAGACAGCAAAAAAGCATTAAAAGGCGTAGTCTTTGAAGTGCAAGATGAAGCTGGAACAGTAGTAAAAGAAGTAAAAACAGATAAAGATGGTAAAGCAAAAATCTCAGATCTATTAGTAGGAAAGTACAAATTAGTAGAGAAAGAAAGTTTACCAGGCTATAAGAAACTAACAGATCCAGTATTATTTGAAATCAAAAAAGGCATGACTAAAGTTCTATCATTAAAAATAGAAAATGAAATGGTAGATACGGGAAATGTAGAGATAACAAAGATAGATAAAGATAATAAAGCACCGTTAGCGGGAGTAACATTCATCGTCCAAGATGAAAAGGGTAATGAAGTTACAAAAGTAACGACAGATAAAGATGGAAAAGCAAATGTTTCAGATTTACCTGTAGGAAAGTATGAATTAGTAGAGGTAGAAAGTTTGCCTGGTTATAAAAAACTAGAAAAACCAGTATCATTTGAAATCAAAAAAGGCATGACCGAGGTTCTATCATTAAAAGTAGAAAATGAAATGGTGGATACAGGGAATGTAGAGATAACAAAAATAGATAAAGATAGTAAAGCTCCATTAGAAAATGTTGTATTTGAAGTACGTGATTTAAAAGGAAAAGTAGTTGCGAAAGTAACGACGGATAAAGAAGGAAAAGCAAACGTTTCAGATTTACCTATTGGGAAGTATGAGTTGGTGGAAGTAGAAACACCGGCAGGATACAAACCGCTAGAAAAGCCAATTTCATTCGAAATTGAAAAGGGTAGAGTAACAGCGCTAAAGCTAACAGTAGAAAATGAATTAGTGGATACAGGAAATGTAGAAATTACAAAAGTAGATAAAGAAAATAAAGATGCTTTAGCTGATGCAGTATTTGAGATTCAAGATGAAGCAGGACAAGTAGTCGCTAAAATAACGACAGATAAAAAAGGACAAGCACAAGTTACTAATTTATCCGTCAGCACATACAAGTTAGTAGAAGTAAAGGCACCAAAAGGGTATAAACAATTAGTAGATCCGATTACTTTCCAAATTGAAAAAGGCATGACAAAATCTCTTGCTTTAACAGTAGAAAACGAAATGTTAGACAAGGGAAATGTGGAAGTAACAAAAGTAGATAAAGATAGTCAAAAAGTATTAGAAGGCGTAGTCTTCGAAGTTCAAGACGACAAAGGCAAAGTAGTAACAGAAGTAACGACAGATAAAGAAGGAAAAGCAAACGTTTCAGATCTATCAGTAGGAAAATACAAATTAGTAGAGACGAAGAGCTTACCAGGTTACAAAAAATTAACAGAACCAGTATCATTCGAAATCAAAAAAGGTATGACGAAAGTTTTATCATTGAAAGTAGAGAATGAACAGTTAGATAAAGGTTCAGTAGAAATTACAAAAGTGGACAAAGAAAGTGGCGCAGTATTAGCGGGCGTAACATTCGAAGTGCAAGATGAAAAAGATAAAGTAGTAACAAAAGTAAAGACAGATAAAGAAGGAAAAGCAAACGTTTCCGATTTATCAGTAGGGAAATATAAGCTAGTAGAGGTAGAAAGCTTACCAGGATATAAAAAATTAGCAAAACCAGTATCGTTTGAAATTAAAAAAGGCATGACAGAAGTCTTATCACTAAAAGTAGAGAATGAACAGTTAGATAAAGGTTCAGTAGAAATTACAAAAGTGGACAAAGACAGCCAGAAGGCATTAGAAGGCGTAACATTCGAAGTGCAAGATGAAAAAGGCAAAGTAGTAACGAAAGTAACAACAGATAAAGAAGGAAAAGTAAAAATTTCAGATCTATCTGTAGGAAGTTACAAACTAGTAGAAGTAGAGAATTTACCAGGCTACAAAAAATTAACAGAGCCAGTATCATTCGAAATTAAAAAGGGTATGACAGAAGTCTTATCATTAAAAGTAGAGAATGAACAGTTAGACAAAGGTTCAGTAGAAATCACAAAAGTAGATAAAGATAGTCAAAAAGTATTAGAAGGCGTAGTCTTCGAAGTACAAGATGAGCAAGGCAAAGTAGTAACGGAAGTAAAAACAGATAAAAATGGTAAAGCAAAAATCTCAGACTTATCTGTAGGAAAGTACAAATTAGTAGAGAAAGAAAGCTTACCAGGTTACAAAAAATTAACAGAACCAGTATCGTTTGAAATCAAAAAAGGAATGACAGAAGTTCTATCATTAAAAATAGAAAATGAAATGGTAGATACGGGAAATGTAGAAATTACAAAAATAGATAAAGATAATAAGGCACCGTTAGCAGGTGTAGTCTTTGAAGTTCAAGACGACAAAGGCAAGGTAGTAACGAAAGTAACGACAGATAAAGCTGGAAAAGCAACAGTTTCAGATTTATCAGTAGGAAAGTACAAGCTAGTAGAAGTAGATAGTTTACCAGGCTACAAAAAATTAGAAAAGCCTGTACCATTTGAAATTAAAAAAGGTATGACAAAATCTTTAACGTTCACTGTAGAAAATGAAATGGTAGATACGGGGAATGTAGAAATCACAAAAATAGATAAAGACAGTAAAGCTCGATTAGAAAATGTTGTATTTGAAGTACGTGACTCAAAAGGAAAAGTAGTTGCAAAAGTAACAACGGATAAAGAAGGAAAAGCAAACGTTTCAGATTTATCTATTGGAAAGTATGAGTTAGTAGAAGTAGAAACACCGGCAGGATACAAGCCACTAGAAAAGCCAGTTTCATTCGAAATTGAAAAAGGTAGAGTTACAGCATTACAATTGACTGTAGAAAATGAATTAGTGGATACAGGAAATATAGAAATTACAAAAGTAGATAAAGAAAATAAAGATGCTTTAGCTGATGCAGTCTTTGAAATTCAAGATGCAGCAGGACAAGTAGTCGCTAAAATAACGACAGATAAAAAAGGACAAGCACAAGTTACTAATTTATCAGTTGGCACATACAAGTTAGTAGAAGTAAAAGCACCAAAAGGATATAAGCAATTGGTAGATCCGATTACTTTCCAAATTGAAAAAGGCATGACAAAATCTCTTGCTTTAACAGTAGAAAATGAAATGTTAGACAAGGGAAATGTAGAAGTAACAAAAGTAGATAAAGATAGCCAAAAAGTATTAGAAGGCGTAGTCTTCGAAGTACAAGATGAGCAAGGCAAAGTAGTAACAGAAGTAACGACAGATAAAGAAGGAAAAGCAAACGTTTCAGATCTATCAGTAGGAAAATACAAATTAGTAGAGACGAAGAGCTTACCAGGTTATAAGAAGTTAACGGAACCAGTATCATTCGAAATTAAAAAAGGTATGACGAAAGTCTTATCATTGAAAGTAGAGAATGAACAGTTAGACAAAGGTTCAGTGGAGATTACAAAAATGGCTGCTGAAAGCAAGGAAGTCTTATCAGGAGCTGTGTTTGAAGTTCATGATGAAAAGGGAAAAGTAGTAGTGAAAGTAACAACAGATAAAGGTGGGAAGGCAAAAATCGCAGATCTATCTGTAGGTAACTACACACTAGTAGAAGTAGAAGCACCAAAAGGATATGAAAAATTAACTAATCCAATTCCATTTGAAATTACAAATGGAATGATAAATGCAGTTCAATTAGAAGTATTAAACAAATTGAATCATTTAGCACCACCAGGTCCAGAAACACCAGATCCAGAAAAACCTGGAACACCAGATCCAGAAAAGCCTGGAACACCAGATCCAGAAAAACCTGGAACACCGAATCCAGAAAAACCTGGAACACCAGATCCAGAAAAACCTGGAACACCGAATCCAGAAAAACCTGGAACACCAGATCCAGAAAAGCCTGGAACACCGAATCCAGAAAAACCTGGAACACCAGATCCAGAAAAACCTGGAACACCGAATCCAGAGAAACCTGGAACACCGAATCCAGAAAAATCGGAAAAAGAATTACCGAAGACAGGGCAGAAAATGCCTGTGGAACCATATATGGGAGCACTTCTTGTAATGATGAGTTTTGGATTATTCGTATTAGGTAGAAAACAGCAGAGATAA

**Supplementary Fig. S1**. **A** Gene organization of the region encoding CbpA in *B. thuringiensis* 407 (Ref Seq accession number NC_018877.1). The genes flanking *cbpA* are represented by the suffix of the given locus tag, in which the locus tag prefix «BTB_» has been omitted for clarity. Bc2 indicates the class I c-di-GMP responsive riboswitch located upstream of *cbpA*. **B** The sequence corresponding to the class I c-di-GMP riboswitch Bc2, detected by Riboswitch Scanner in the upstream region of *cbpA* in *B. thuringiensis* 407, is marked in red. The start codon of *cbpA* is underlined and marked in bold. The predicted secondary structure of the riboswitch based on minimum free energy calculations is shown. The minimum free energy of the predicted secondary structure is -16.90 kcal/mol. **C** Sequence of the *cbpA* gene (locus tag BTB_RS05575 in NC_018877.1: 1089279-1095841) in *B. thuringiensis* 407, as obtained by resequencing of PCR products bridging the gap spanning two contigs in ACMZ00000000, and correcting the frameshift present in *cbpA* (BTB_RS05575) in NC_018877.1.


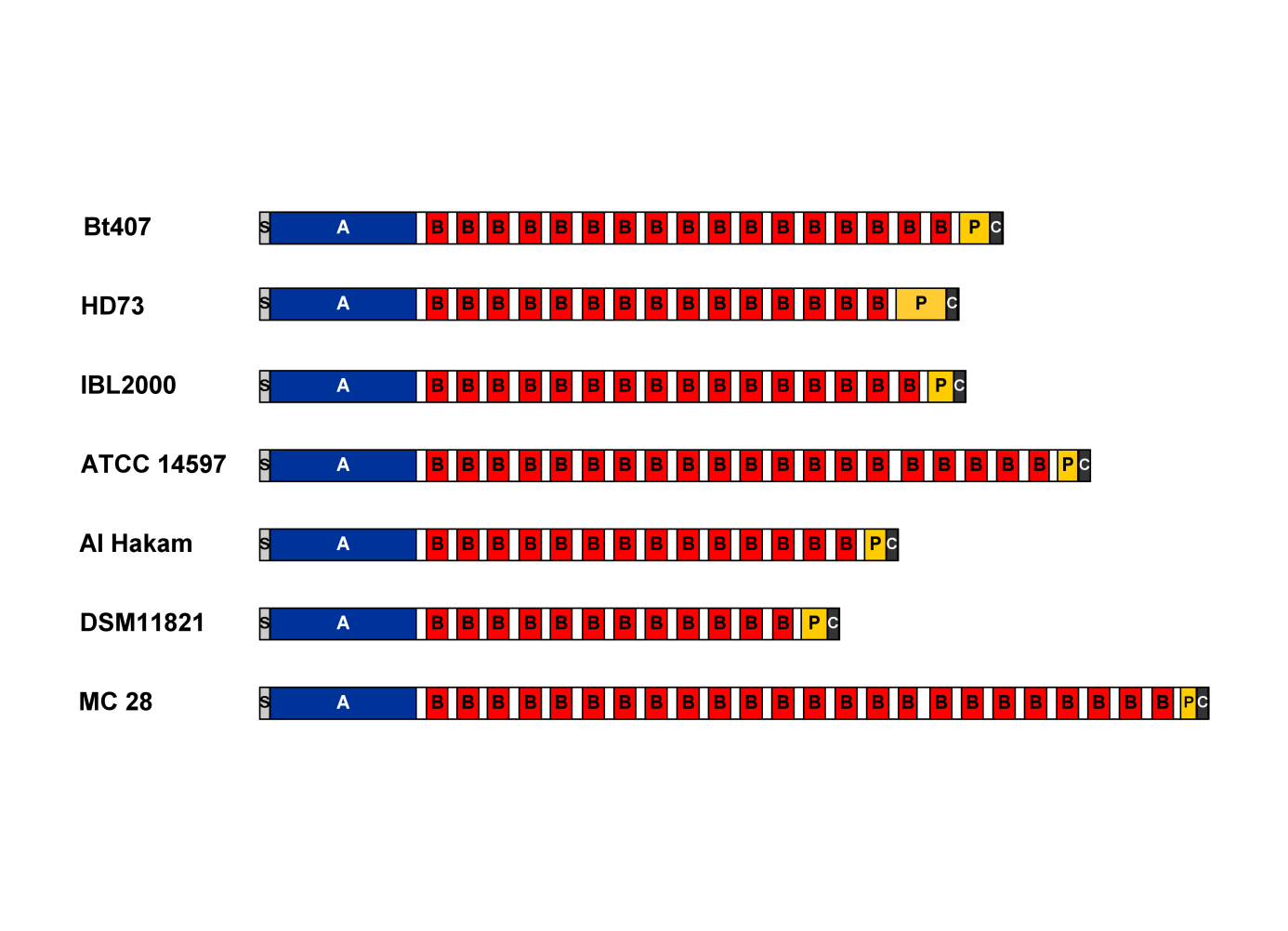


**Supplementary Fig. S2.** Schematic representation of the domain organization of CbpA proteins from a selection of strains belonging to the *B. cereus* group, in comparison to CbpA from *B. thuringiensis* 407 (Bt407, top), showing the signal peptide sequence (S), the predicted subdomains of the A-region (N1, N2, and N3), the Cna_B-type domain repeats of the B-region (B), the proline-rich repeat region (P), and the cell wall sorting signal containing the LPXTG sortase substrate motif (C).

**A**

**B**

**
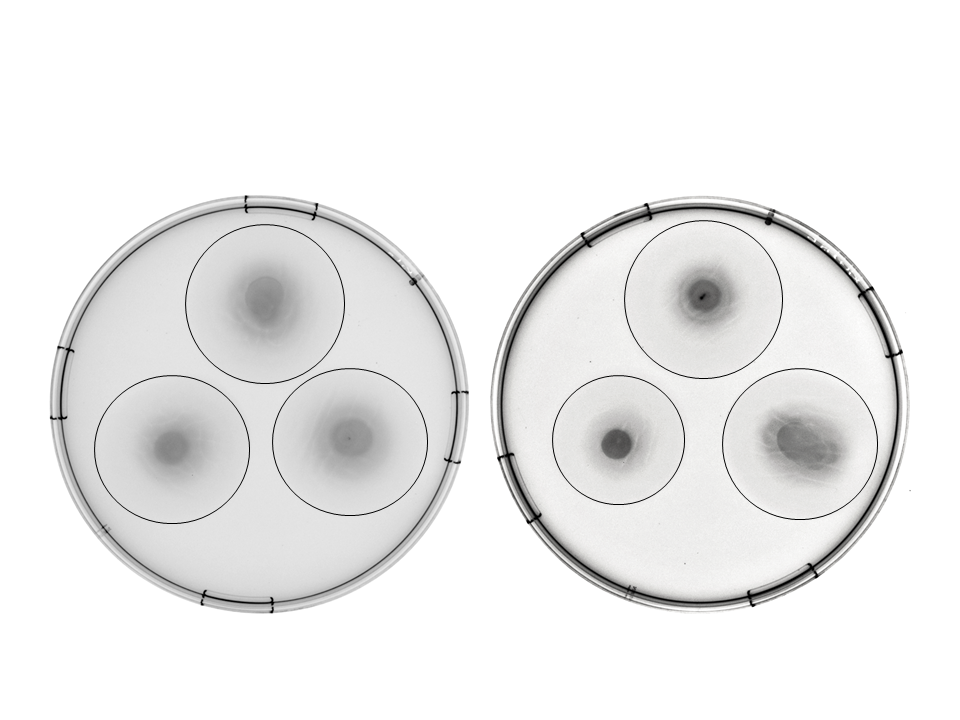

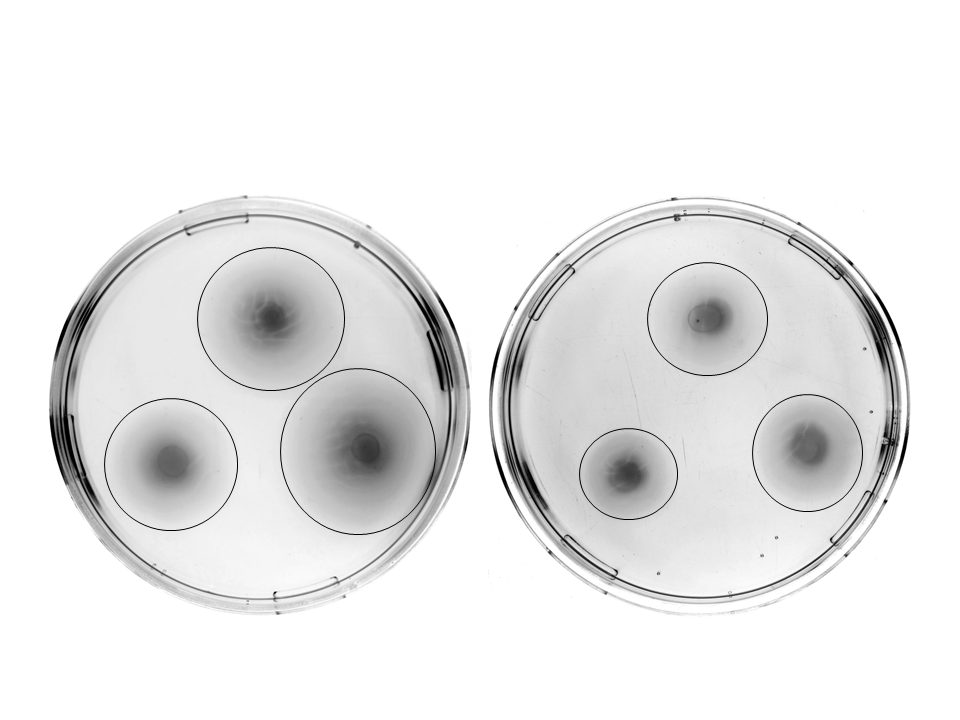
**

**DD**

**C**

**Supplementary Fig. S3.** Influence of CbpA on swimming motility in *B. thuringiensis* 407 was assessed after growth for 7 h on LB plates containing 0.3 % agar. The swimming zones are marked with circles for better visualization. **A**,**B** Prior to spotting on swimming plate bacteria were grown in LB medium to OD_600_ ≈ 0.2. **C**, **D** Prior to spotting on swimming plate bacteria were grown in LB medium for 16 h to reach the stationary growth phase. **A** and **C** show *B. thuringiensis* 407 wild type (Bt407) (top), the *cbpA* deletion mutant (Bt407Δ*cbpA*)(right) and the complementation strain (Bt407Δ*cbpA* pHT304-Pxyl-*cbpA*) (left). **B** and **D** show the empty vector control strain (Bt407 pHT304-Pxyl) (top), CbpA overexpression strain (Bt407 pHT304-Pxyl-*cbpA*) (left) and complementation strain (Bt407Δ*cbpA* pHT304-Pxyl-*cbpA*) (right).
